# Analysis of the sucrose synthase gene family in tobacco: structure, phylogeny, and expression patterns

**DOI:** 10.1007/s00425-015-2297-1

**Published:** 2015-04-19

**Authors:** Zhong Wang, Pan Wei, Mingzhu Wu, Yalong Xu, Feng Li, Zhaopeng Luo, Jianfeng Zhang, Ang Chen, Xiaodong Xie, Peijian Cao, Fucheng Lin, Jun Yang

**Affiliations:** China Tobacco Gene Research Center, Zhengzhou Tobacco Research Institute of CNTC, Zhengzhou, 450001 China; China School of Life Sciences, Xiamen University, Xiamen, 361102 Fujian China

**Keywords:** Phylogeny, Stress, Sucrose, Tobacco

## Abstract

**Provide an evolutionary and an empirical molecular genetic foundation of the Sus gene family in tobacco and will be beneficial for further investigations of Sus gene functions**

Sucrose synthase (Sus) has been well characterized as the key enzyme participating in sucrose metabolism, and the gene family encoding different Sus isozymes has been cloned and characterized in several plant species. However, scant information about this gene family is available to date in tobacco. Here, we identified 14, 6, and 7 *Sus* genes in the genomes of *Nicotiana tabacum*, *N.sylvestris* and *N.tomentosiformis*, respectively. These tobacco Sus family members shared high levels of similarity in their nucleotide and amino acid sequences. Phylogenetic analysis revealed distinct evolutionary paths for the tobacco *Sus* genes. *Sus1–4*, *Sus5*, and *Sus6*–*7* originated from three *Sus* precursors, respectively, which were generated by duplication before the split of monocots and eudicots. There were two additional duplications, before and after the differentiation of the Solanaceae, which separately gave rise to *Sus3/4* and *Sus1*/*2*. Gene exon/intron structure analysis showed that the tobacco *Sus* genes contain varying numbers of conserved introns, resulting from intron loss under different selection pressures during the course of evolution. The expression patterns of the *NtSus* genes differed from each other in various tobacco tissues. Transcripts of Ntab0259170 and Ntab0259180 were detected in leaves at all tested developmental stages, suggesting that these two genes play a predominant role in sucrose metabolism during leaf development. Expression of Ntab0288750 and Ntab0234340 were conspicuously induced by low temperature and virus treatment, indicating that these two isozymes are important in meeting the increased glycolytic demand that occurs during abiotic stress. Our results provide an evolutionary and an empirical molecular genetic foundation of the *Sus* gene family in tobacco, and will be beneficial for further investigations of *Sus* gene functions in the processes of tobacco leaf development and tobacco resistance to environmental stresses.

**Electronic supplementary material:**

The online version of this article (doi:10.1007/s00425-015-2297-1) contains supplementary material, which is available to authorized users.

## Introduction

Sucrose is essential for the plant life cycle. It is mainly produced by photosynthesis in leaves, and is exported to sink tissues that serve as carbon and energy sources for growth processes and for the synthesis of storage reserves (Lunn and Furbank 1999; Chen et al. 2012). When suffering low temperature or drought stress, plant cells can accumulate sucrose to stabilize membranes and proteins. Further, sucrose is thought to supply energy to ramp up metabolism when such stress ceases (Yang et al. 2001; Strand et al. 2003). It has also been shown that sucrose acts as a signal in plants to modulate the expression level of genes encoding enzymes, storage proteins, and transporters (Ciereszko et al. 2001; Stitt et al. 2002; Vaughn et al. 2002; Zourelidou et al. 2002). Moreover, sucrose participates in the regulation of several developmental processes, such as cell division (Gaudin et al. 2000), flowering induction (Ohto et al. 2001), vascular tissue differentiation (Uggla et al. 2001), seed development (Iraqi and Tremblay 2001), and the accumulation of storage products (Rook et al. 2001). Thus, the study of the metabolism of sucrose is central in understanding myriad aspects of plant physiology.

Sucrose synthase (Sus) and invertase (Inv) are the two key enzymes that cleave sucrose before its transfer to sink organs. Inv catalyzes the hydrolysis of sucrose into fructose and glucose. Sus catalyzes the conversion of UDP (uridine diphosphate) and sucrose into UDP-glucose and fructose (Schmalstig and Hitz 1987; Kleczkowski et al. 2011). Both Sus and Inv supply energy for phloem loading (Martin et al. 1993; Coleman et al. 2009), and Sus also participates in distributing carbon resources into various pathways that are necessary for the metabolic and storage physiology of the plant cell (Haigler et al. 2001; Ruan et al. 2008). For instance, previous studies have demonstrated that Sus cleavage activity is closely related to the strength of various starch storing sinks, such as potato tubers, pea embryos, and maize kernels (Zrenner et al. 1995; Chourey et al. 1998; Barratt et al. 2001). Moreover, low temperature and drought stress are known to induce the expression of *Sus* genes in *Hevea brasiliensis* (para rubber tree), suggesting a positive role for Sus in stress resistance (Xiao et al. 2014).

Previous studies have demonstrated that Sus isoforms are encoded by a small multi-gene family. The number of members in the *Sus* gene family differs among the plant species examined to date. For instance, the maize and pea genomes contain three *Sus* genes (Barratt et al. 2001; Duncan et al. 2006), while there are six distinct *Sus* genes in Arabidopsis, rice, and *Lotus japonicas* (Baud et al. 2004; Horst et al. 2007; Hirose et al. 2008). Seven *Sus* genes have been identified in poplar (An et al. 2014). Diploid cotton genomes (*Gossypium arboreum* L. and *G. Raimondii* Ulbr.) each contain eight *Sus* genes, while the tetraploid cotton genome (*G. Hirsutum* L.) contains fifteen members, representing the largest *Sus* family observed to date (Zou et al. 2013). It is also known that the functions and expression patterns of *Sus* genes are divergent at different development stages in the same plant. For example, the pea genes *Sus1*, *Sus2,* and *Sus3* are expressed predominately in, respectively, developing seeds, leaves, and flowers. Furthermore, *Sus2* and *Sus3* are not able to compensate for the activity of *Sus1* in root nodules or seeds (Barratt et al. 2001). *Sus1* is expressed ubiquitously in maize and functions mainly in starch synthesis, while the expression of maize *Sh1* is abundant in developing endosperm tissue and may promote cell wall synthesis (Duncan et al. 2006). Arabidopsis *Sus* genes can be divided into three groups with distinct but partially overlapping expression profiles; the function of the Arabidopsis *Sus* genes is also distinct, according to studies of loss-of-function mutants (Bieniawska et al. 2007). Tissue-specific and developmental-dependent expression patterns of *Sus* genes have also been observed in many other plant species including *L. japonicus*, rice, and citrus (Wang et al. 1999; Komatsu et al. 2002; Horst et al. 2007; Hirose et al. 2008). The differential expression patterns of *Sus* genes might indicate specialized functions. Although the *Sus* gene family has been extensively studied in a few plant species such as Arabidopsis, rice, cotton, and poplar, the *Sus* genes in tobacco have not.

Tobacco is an economically important plant throughout the world. Natural tobacco leaves contain about 20 % sugar by weight, including glucose, fructose, and sucrose (Davis and Mark 1999; Talhout et al. 2006). Sucrose, fructose, glucose, and inverted sugar (a mixture of fructose and glucose) are frequently used as a cigarette additive; these are thought to function as humectants, and to improve both casing and flavor (Davis and Mark 1999; Seeman et al. 2003; Talhout et al. 2006). Up to 13 % (w/total w) of sugars and sweeteners are added to tobacco during the manufacturing process (Davis and Mark 1999; Fowles and Bates 2000; Smith et al. 2002; Seeman et al. 2003). Sugars can improve the experience of smoking of cigarettes through neutralizing the throat impact and harsh taste of smoke, but sugars are also known to generate acetaldehyde, which is addictive to rodents (Talhout et al. 2006). In addition to being hydrolyzed into monosaccharide moieties by Inv, sucrose can also be used to produce UDP-glucose. It has been demonstrated that an 88-kD Sus polypeptide can catalyze the synthesis of UDP-glucose in the inner layer of tobacco pollen tubes. UDP-glucose is required as a metabolic precursor for the biosynthesis of both cellulose and callose. Both of these polymers are necessary components of the inner layer of tobacco pollen tubes (Diana et al. 2008). As the functions of the other tobacco *Sus* genes are unknown, it is necessary to identify and characterize the *Sus* gene family in tobacco to explore their functions and evolutionary relationships.

In the present study, we identified 27 *Sus* genes in an allotetraploid (*Nicotianatabacum*) and two diploid (*N.sylvestris* and *N.tomentosiformis*) tobacco species. We then focused on the locations of each member of the tobacco *Sus* gene family in their respective genomes, their evolutionary relationships, their intron/exon organization, their tissue- and developmental-dependent expression patterns, and their potential roles in responses to environment stresses. Our results provide a foundation for further investigations into the specific functions of each tobacco *Sus* gene, particularly during leaf development and maturation.

## Materials and methods

### Plant materials and growth conditions

*Nicotianatabacum* L. (Honghua Dajinyuan) was used in the analysis of the expression profiles of the *Sus* genes. Tobacco seeds, which are kept by our own lab, were germinated and maintained in pots under normal conditions (16 h light at 28 °C day, 23 °C night) until flowering, and then plant roots, stems, leaves, buds, sepals, stamens, pistils, and seeds were collected for RNA extraction. Tobacco seedlings with 9–11 true leaves were transplanted to open fields for continued growth. Leaves from different development stages were harvested for further analysis of the expression of *Sus* genes.

For the stress treatment experiments, tobacco seeds were germinated on 1/2 MS medium in darkness after soaking and sterilizing. Seedlings were then transplanted to vermiculite for continued growth to the six-leaf stage. Uniform seedlings were chosen, washed with deionized water, and transferred to a 1/3 concentration of Hoagland solution for 1 week prior to use in the stress experiments. For the drought treatment, seedlings were cultivated in a solution containing 20 % (w/v) PEG6000 for 2 days. For the low-temperature treatment, seedlings were kept at 0 °C in an illuminated incubator for 24 h. Tobacco mosaic virus was inoculated onto six leaves for 10 days. After treatment, plant materials were collected and immediately frozen in liquid nitrogen and stored at −80 °C prior to RNA extraction.

### Phylogenetic and gene structure analyses

The Solanaceae Sus sequences were obtained from http://solgenomics.net/. The Sus sequences of other plant species were collected by searching the NCBI GeneBank database using ‘sucrose synthase’ as a query keyword. DNAMAN (version 6.0) and Clustal X (version 1.83) were used to perform the multiple alignments of the *Sus* nucleotide and deduced amino acid sequences, respectively, with default gap penalties. The phylogenetic tree of the Sus amino acid sequences was constructed with MEGA 5.0 using the neighbor-joining algorithm.

We aligned each *Sus* cDNA sequence and its corresponding genomic DNA sequence to identify the exon/intron locations. Protein sequence motifs were predicted using the multiple EM for motif elicitation program (MEME, http://meme.nbcr.net/meme3/mme.html). Motifs identified with MEME (≤1E−100) were further queried in the InterPro database (http://www.ebi.ac.uk/interpro/;jsessionid=F412A1E32D81ECA4EBDB9A250D55D32E) (Jones et al. 2014).

### RNA extraction and cDNA preparation

Total RNA was extracted using a SuperPure Plantpoly RNA Kit (Gene Answer). RNase-free DNase I (Gene Answer) was used in the extraction process to remove DNA contamination. Both the concentration and the quality of the RNA samples were evaluated with a Nanodrop 2000 instrument (Thermo). 1 μg total RNA was used to synthesize first strand cDNA using Reverse Transcriptase M-MLV (TAKARA) with random primers. After reverse transcription, the concentrations of the cDNA samples were evaluated with the Nanodrop 2000 instrument, and then diluted to 100 ng/μl.

### RT-PCR and RT-qPCR

The gene specific primers used in the RT-PCR and the RT-qPCR experiments are listed in Suppl. Table S1. RT-PCR was performed using TAKARA Taq polymerase in a heated lid thermal cycler (Biometra). The PCR cycling program was as follows: 95 °C for 5 min, 26–30 cycles of 30 s at 94 °C, 30 s at 55 °C or 60 °C, and 30 s at 72 °C. RT-qPCR amplification reactions were performed using an iCycler iQ thermo cycler (Bio–Rad) and a SYBR Green kit (Bio–Rad). The PCR program was as follows: 95 °C for 5 min, 40 cycles of 30 s at 94 °C, 30 s at 60 °C, signal acquisition, and then a final melting curve of 65–95 °C. The expression levels of the *NtSus* genes in leaves from different development stages were standardized to the expression level of the *Ls25* gene at each corresponding stage.

## Results

### Identification of *Sus* genes in tobacco

We first searched the *N.tabacum* genome in the China tobacco genome database v 2.0 (data not shown) using ‘sucrose synthase’ as the query key word, and thusly obtained 16 putative *Sus* genes. We then performed several additional BLAST searches of the *N.tabacum* database using the amino acid sequences of Arabidopsis Sus proteins as the query sequences. We concatenated all the search records and made an alignment of their amino acid sequences. We excluded short and/or low identity sequences, and finally retained 14 *NtSus* genes. As shown in Table 1, the genomic DNA size of these 14 *NtSus* genes varied from 3.5 to 15 kb, but the cDNA sizes were all quite similar. The putative polypeptides of these *NtSus* genes contained between 642 and 909 amino acids (molecular weights ranging from 72.71 to 103.27 kDa) with estimated isoelectric points between 5.7 and 7.85. These predicted molecular features of the NtSus enzymes were similar to those of previously characterized Sus isozymes from other plant species. We identified and characterized 6 and 7 *Sus* genes from the *N.sylvestris* and *N.tomentosiformis* genomes (Table 1), respectively.

**Table 1:** Characteristics of sucrose synthase genes in tobacco

	Gene ID	Gene model ID	gDNA size (bp)	CDS size (bp)	Amino acid size	MW (kDa)	pI
*Nicotiana tabacum*	Ntab0023590	Ntab0023590.1	3489	1929	642	72.71	5.7
	Ntab0195900	Ntab0195900.1	4532	2418	805	92.5	5.98
	Ntab0234340	Ntab0234340.1	4121	2667	888	100.41	6.86
	Ntab0259170	Ntab0259170.1	4163	2418	805	92.57	6.09
	Ntab0259180	Ntab0259180.1	4379	2412	803	91.17	6.06
	Ntab0288750	Ntab0288750.1	14,998	2433	810	92.46	6.32
	Ntab0298870	Ntab0298870.1	4053	2418	805	92.63	6.03
	Ntab0298880	Ntab0298880.1	3926	2412	803	91.2	5.91
	Ntab0385170	Ntab0385170.1	3922	2730	909	103.27	7.85
	Ntab0452620	Ntab0452620.1	8323	2400	799	91.28	6.44
	Ntab0594750	Ntab0594750.1	3739	2616	871	99	6.48
	Ntab0679080	Ntab0679080.1	3489	1929	642	72.71	5.7
	Ntab0784850	Ntab0784850.1	4134	2475	824	92.7	8.06
	Ntab0820630	Ntab0820630.1	4079	2418	805	92.57	5.98
*Nicotiana sylvestris*	Nsyl0248340	Nsyl0248340.1	4505	2418	805	92.55	5.93
	Nsyl0289930	Nsyl0289930.1	4229	2295	764	87.65	6.04
	Nsyl0289940	Nsyl0289940.1	4360	2412	803	91.16	6.06
	Nsyl0308810	Nsyl0308810.1	8378	2433	810	92.46	6.24
	Nsyl0056400	Nsyl0056400.1	4121	3537	1178	100.41	6.86
	Nsyl0090300	Nsyl0090300.1	17,939	2667	888	133.37	5.95
*Nicotiana* *tomentosiformis*	Ntom0116740	Ntom0116740.1	4065	2418	805	92.46	6.03
	Ntom0289900	Ntom0289900.1	4053	2418	805	92.63	6.03
	Ntom0008050	Ntom0008050.1	3163	1656	551	62.73	5.99
	Ntom0289890	Ntom0289890.1	3920	2412	803	91.17	5.97
	Ntom0141700	Ntom0141700.1	14,039	2433	810	92.51	6.32
	Ntom0061210	Ntom0061210.1	22,937	2937	978	11.03	6.51
	Ntom0041210	Ntom0041210.1	3815	2652	883	99.92	6.55

Identities between the amino acid sequences of the 14 NtSus proteins were calculated with the DNAMAN algorithm. As shown in Table 2, the NtSus sequences could be divided into seven pairs that each had high levels of similarity (higher than 86.15 %), a result suggesting that one *Sus* gene has two putative paralogs in the tetraploid tobacco genome. Alignment between the coding sequences of the 14 *NtSus* genes also confirmed this hypothesis. Moreover, we performed intra- and inter-species sequence alignments between the amino acid sequences and coding sequences of the six NsylSus and the seven NtomSus, and found six pairs of them shared much higher sequence identities (82.72–99.01 % at the amino acid level, 82.26–97.99 % at the nucleotide level) in the inter-species alignment (Suppl. Table S2). We named the seven pairs of *Sus* genes as *Sus1*–*7* in *N.tabacum*, and identified their putative orthologous genes in *N.sylvestris* and *N.tomentosiformis* by comparing the identities of the Sus sequences (Table 3). Both of the diploid genomes contained one ortholog for each *Sus* gene, with the exception of *Sus4* in *N.sylvestris*. These homologous relationships were also confirmed by subsequent phylogenetic analysis (Suppl. Fig. S1).

**Table 2:** Identity matrix of predicted NtSus amino acid sequences and their coding sequences

	Amino acid identity													
	Ntab0023590	Ntab0679080	Ntab0259180	Ntab0298880	Ntab0259170	Ntab0298870	Ntab0195900	Ntab0820630	Ntab0288750	Ntab0452620	Ntab0234340	Ntab0784850	Ntab0385170	Ntab0594750
Nucleotide identity														
Ntab0023590		*100*	63.76	64.13	49.44	49.32	50.19	49.69	42.66	42.29	32.19	19.47	33.41	35.21
Ntab0679080	*100*		63.39	64.13	49.44	49.32	49.81	49.69	42.66	42.29	32.19	19.47	33.41	34.63
Ntab0259180	67.99	68.03		*98.01*	71.73	71.23	72.35	72.1	61.13	60.02	45.76	40.29	46.89	49.54
Ntab0298880	68.16	68.33	*97.14*		70.86	70.62	71.98	71.73	60.89	59.78	45.65	40.29	46.6	49.43
Ntab0259170	52.97	52.97	70.3	70.13		*98.76*	93.66	93.54	70.99	69.63	46.37	40.8	47.97	50.29
Ntab0298870	52.68	52.56	69.8	69.52	*95.7*		93.42	93.29	70.99	69.63	46.48	40.91	47.86	50.17
Ntab0195900	52.68	52.76	69.97	70.16	87.88	87.84		*99.63*	70.74	69.51	46.59	41.47	47.75	50.17
Ntab0820630	52.85	52.85	69.54	69.67	88.01	87.88	*96.15*		70.62	69.63	46.48	41.36	47.42	49.83
Ntab0288750	49.47	49.22	64.37	64.5	68.68	68.8	68.73	68.64		*97.28*	48.68	42.81	50.27	52.58
Ntab0452620	48.69	48.32	63.47	63.47	67.28	67.49	67.38	67.37	*96.75*		48.72	43	49.51	51.78
Ntab0234340	38.84	39.16	51.01	51.12	51.02	51.41	51.37	51.17	52.54	52.92		*86.15*	65.83	65.64
Ntab0784850	37.92	37.92	44.09	47.92	48.44	48.39	48.92	48.89	50.11	50.39	*89.46*		57.94	59.47
Ntab0385170	39.37	39.29	51.39	51.41	52.7	52.56	52.36	51.94	53.55	52.95	67.31	62.98		*91.57*
Ntab0594750	42.11	42.08	54.3	54.51	55.08	55.1	54.85	54.47	56.06	55.64	66.06	62.76	*92.89*

**Table 3:** Sus orthologs among *Nicotiana tabacum*, *Nicotiana* *sylvestris,* and *Nicotiana* *tomentosiformis*

Protein symbol	*Nicotiana tabacum*	*Nicotiana sylvestris*	*Nicotiana tomentosiformis*
Sus1	Ntab0195900	Nsyl0248340	Ntom0116740
	Ntab0820630		
Sus2	Ntab0259170	Nsyl0289930	Ntom0289900
	Ntab0298870		
Sus3	Ntab0259180	Nsyl0289940	Ntom0289890
	Ntab0298880		
Sus4	Ntab0023590	NO	Ntom0008050
	Ntab0679080		
Sus5	Ntab0288750	Nsyl0308810	Ntom0141700
	Ntab0452620		
Sus6	Ntab0385170	Nsyl0090300	Ntom0041210
	Ntab0594750		
Sus7	Ntab0234340	Nsyl0056400	Ntom0061210
	Ntab0784850		

### Location of *Sus* genes in tobacco genomes

**Fig. 1:**
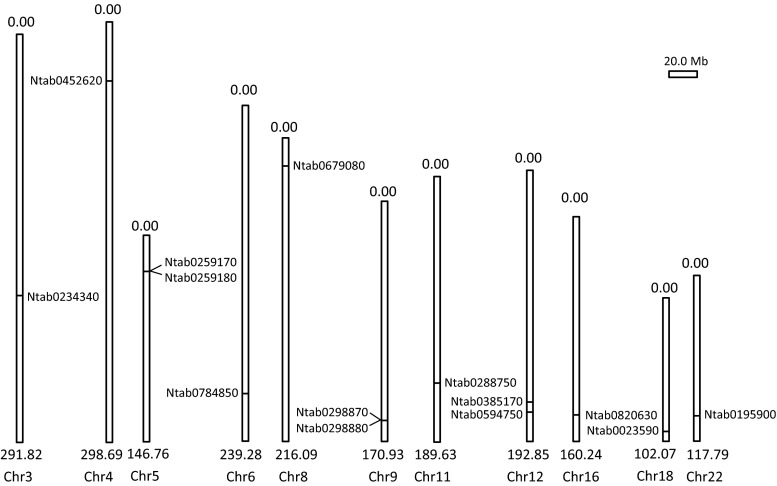
Location of *Sus* gene family members on *Nicotiana tabacum* chromosomes. *Scale* represents a 20.0-Mb chromosomal distance. Chromosome *sizes* (Mb) and *numbers* are indicated at the *bottom end* of each scaffold

We obtained physical maps for each chromosome of the three tobacco species from the China tobacco genome database v 2.0. These maps included the length of the chromosomes, gene numbers, start/end sites of the *Sus* genes. We subsequently drew three simple maps that showed the distribution of the *Sus* genes among the chromosomes in the three tobacco species. As shown in Fig. 1, in *N.tabacum,* 14 *NtSus* genes were located on 11 chromosomes, three of which possessed two genes, while other chromosomes contained only one gene. According to this physical map and the start/end point of each gene on the chromosome obtained from the database, the Ntab0259170/Ntab0259180 and the Ntab0298870/Ntab0298880 pairs were found to have close linkage. As Ntab0259170 and Ntab0298870 are two *Sus2* paralogs, while Ntab0259180 and Ntab0298880 are *Sus3* paralogs (Table 3), it was clear that *NtSus2* and *NtSus3* were closely linked to each other in the *N.tabacum* genome. Moreover, in *N.tomentosiformis* and *N.sylvestris, Sus2* (Ntom0289900, Nsyl0289930) and *Sus3* (Ntom0289890, Nsyl0289940) were also linked (Suppl. Fig. S2 and S3), suggesting that this linkage relationship has been conserved during the evolution of these genomes. The two *Sus6* genes (Ntab0385170 and Ntab0594750) were on the same chromosome, but were not tightly linked; their locations may have resulted from chromosomal exchange during the formation of the tetraploid genome.

### Gene structure and conserved motifs in the tobacco *Sus* gene family

**Fig. 2:**
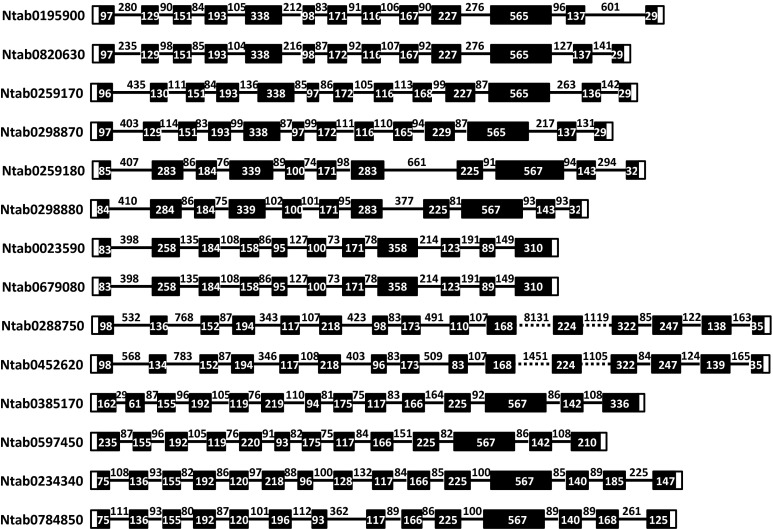
Exon/Intron structural organization of the 14 *NtSus* genes. *Black boxes* denote exons within coding regions, and the *lines* connecting them represent introns. *Numbers* in *boxes* or *above lines* represent the sizes (bp) of corresponding exons or introns, respectively. The 5′ and 3′ untranslated regions (UTRs) are represented by *blank boxes*

**Fig. 3:**
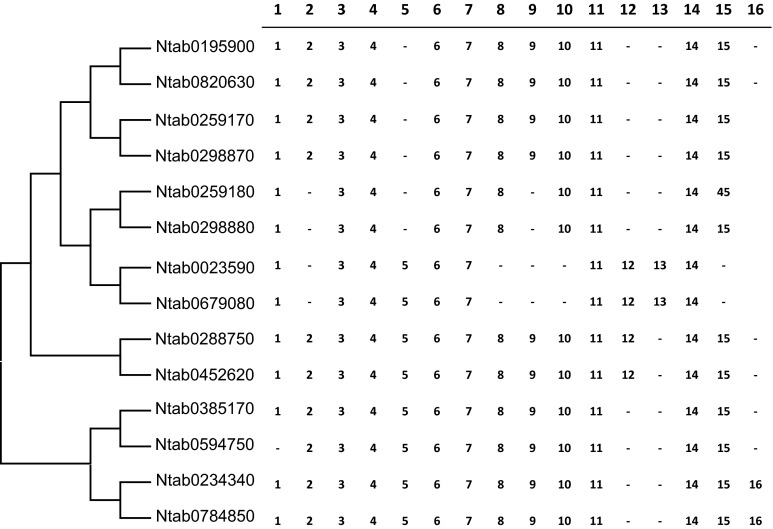
Schematic presentation of the conservation of intron numbers and positions of *Sus* genes from *Nicotiana tabacum*. Sequences are ordered according to the phylogenetic relationship of the corresponding coding regions, as shown on the *left*. Intron positions are indicated in relation to the position of exons. *Dashes* indicate the loss of introns in corresponding positions

To better understand the genesis of *Sus* family genes in tobacco, we analyzed the intron/exon arrangement of each tobacco *Sus* gene. To correctly distinguish intron and exon fragments of *NtSus* genes, we aligned the genomic and corresponding cDNA sequences with DNAMAN software. As shown in Fig. 2, the coding regions of the *NtSus* genes were interrupted by introns of varying sizes. The lengths of these introns varied from 70 to 600 bp, with the exception that two introns in *NtSus5* were longer than 1 kb. Moreover, the numbers of introns were also different among the seven *NtSus* genes. For instance, there were 12 introns in *NtSus1* and *NtSus2*, 10 in *NtSus3* and *NtSus4*, and 14 in *NtSus5* and *NtSus7*. For *NtSus6*, the Ntab0385170 gene contained 13 introns, while the Ntab0597450 gene contained 12. In the diploid tobacco genomes, most *Sus* genes contained the same number of introns as their corresponding orthologs in *N.tabacum*, except for *NsylSus2* and *NtomSus4*, each contained one less intron than *NtSus2* and *NtSus4*, respectively, (Suppl. Fig. S4). We carefully observed the intron positions relative to the conserved exons, and identified 16 putative positions for introns, among which 14 introns (excepting introns 13 and 16) were found in most of the *NtSus* sequences (Fig. 3). The lack of one or more introns, which mainly occurred in the 5th, 9th, 10th, 12th, and 13th intron positions, lead to the formation of larger exons, including exons of 339, 358, and 567 bp in length in some of the *NtSus* genes (Figs. 2, 3).

In addition to our analysis of the nucleotide sequences, we also analyzed the amino acid sequences of the NtSus family. There was a conserved serine residue in the N-terminal regions of all of the NtSus sequences (Suppl. Fig. S1). It has been demonstrated in maize that a Ser/Thr protein kinase can phosphorylate this Ser residue (Huber et al. 1996; Hardin and Huber 2004). Moreover, among the NtSus sequences, we also found two conserved domains that are considered to be characteristic of Sus proteins: a sucrose synthase and a glucosyl-transferase domain. We used MEME to predict the putative motifs in the NtSus sequences, and identified a total of ten distinct motifs. The length, conserved sequence, and predicted molecular function of each motif are listed in Table 4. Most of the motifs were predicted to be involved in sucrose metabolic processes, with the exceptions of motif 6 and 10, the biological significance of which remain to be determined. As expected, the orthologous genes of the three tobacco species contained the same motifs and the same motif arrangement (Suppl. Fig. S5), indicating that these likely have similar functions.

**Table 4:**
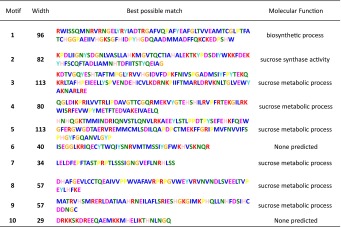
Putative conserved motifs in the amino acid sequences of the 14 NtSus sequences

### Phylogenetic analysis of tobacco *Sus* genes

**Fig. 4:**
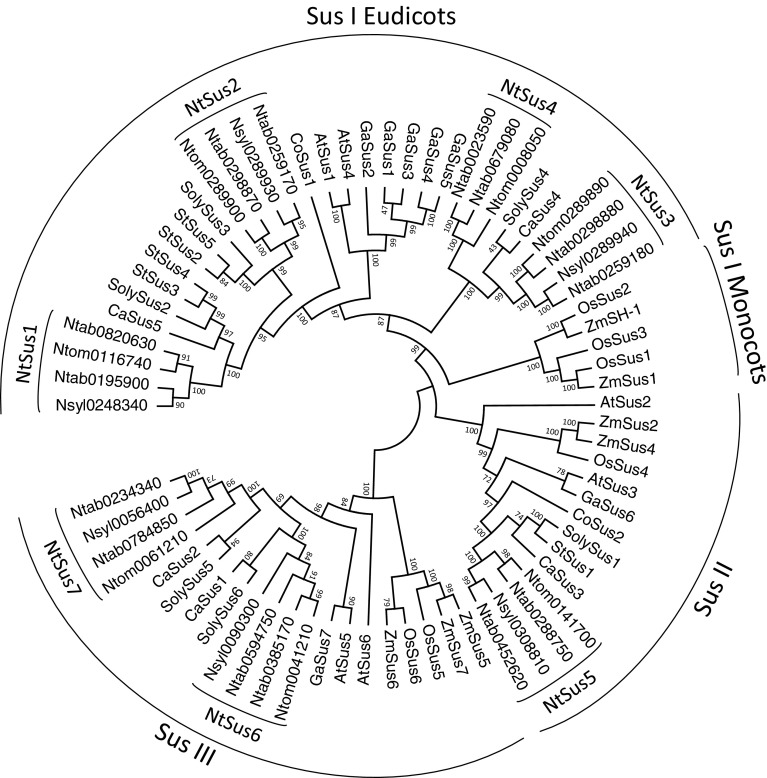
Phylogenetic analysis of tobacco Sus isoforms and other plant Sus homologs. Isozymes and corresponding plant species are: tobacco, Ntab, Nsyl and Ntom (this study); cotton, GaSus1–6 (Chen et al. 2012); *Arabidopsis thaliana*, AtSus1–6 (Baud et al. 2004); rice, OsSus1–6 (Hirose et al. 2008); potato, StSus1–5 (accession number: AY205302, AY205084, STU21129, M18745, STU24088); tomato, Soly1–6 (accession number: Solyc09g098590, Solyc12g009300, Solyc07g042550, Solyc07g042520, Solyc03g098290, Solyc02g081300); pepper, Ca1–5 (accession number: CA02g20320, CA03g23230, CA03g25020, CA07g09080, CA09g14780); coffee Sus1–2 (AM087675; AM087674); maize, ZmSus1, 2, 4, 6, 7 & SH-1 (NCBI accession: NC_024467.1; NC_024459.1, NC_024459.1, NC_024462.1, NC_024463.1, NC_024467.1)

To better understand the evolutionary relationships among the *Sus* genes of tobacco and other plant species, 71 amino acid sequences from 11 species were used to make an alignment using ClustalX. An unrooted tree was constructed based on the alignment using the Neighbor-Joining method implemented in MEGA-5. As shown in Fig. 4, plant *Sus* genes could be divided into four sub-families. Based on several previous analyses with which our results were consistent, we here designated these sub-families as follows: Sus I eudicot group, Sus I monocot group, Sus II group, and Sus III group (An et al. 2014). Most of the tobacco *Sus* genes belonged to the Sus I eudicot group, which included *Sus* genes from other eudicot species including Arabidopsis (*AtSus1* and *4*), *Gossypium arboretum* (*GaSus1*, *3*, *4*, and *5*), potato (*St2*, *3*, *4*, and *5*), tomato (*Soly2, 3,* and *4*), pepper (*CaSus4* and *5*) and coffee (*CoSus1*). There was one tobacco *Sus* gene (*Sus5*) in the Sus II group and two tobacco genes (*Sus6*, *Sus7*) in the Sus III group; no tobacco genes occurred in the Sus I monocot group (Fig. 4). Although the tobacco Sus paralogs shared high sequence similarities, our phylogenetic analysis revealed that diversification has occurred within this family, likely indicating discrete evolutionary histories and diverse biological roles for the members of this gene family in tobacco.

### Differential expression patterns of *NtSus* genes

**Fig. 5:**
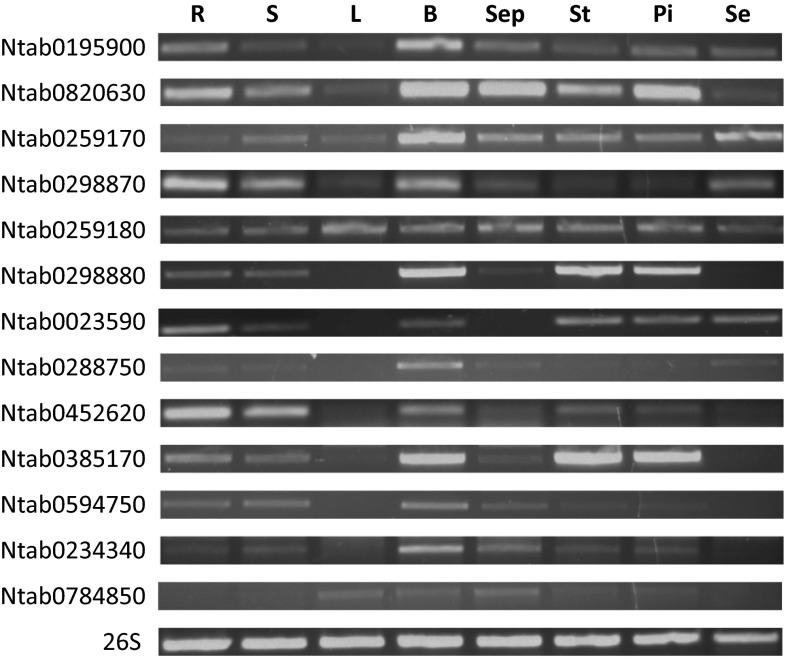
RT-PCR analysis of *NtSus* genes in different tobacco tissues. RNA samples were collected from R (root), S (stem), L (leaves), B (bud), Sep (sepal), St (stamen), Pi (pistil) and Se (seed)

The expression patterns of genes can often partly reveal their likely physiological functions. Therefore, to better understand the possible functions of specific NtSus isoforms in tobacco, we performed tissue-specific expression analysis of *NtSus* genes in roots, stems, leaves, buds, sepals, stamens, pistils, and seeds. As shown in Fig. 5, the expression of Ntab0259170 and Ntab0259180 was detected in all tissues examined, though at different levels. On the contrary, Ntab0288750, Ntab0234340, and Ntab0784850 were mainly expressed in buds and sepals, suggesting probable tissue-specific functions for these three genes. Moreover, transcripts of other *NtSus* genes were detected in most tissues, but each gene was most highly expressed in a particular tissue. For example, the expression of Ntab0195900 was detected in all tissues examined, with the exception of leaves, but it was expressed at much higher levels in buds than in other tissues (Fig. 5).

**Fig. 6:**
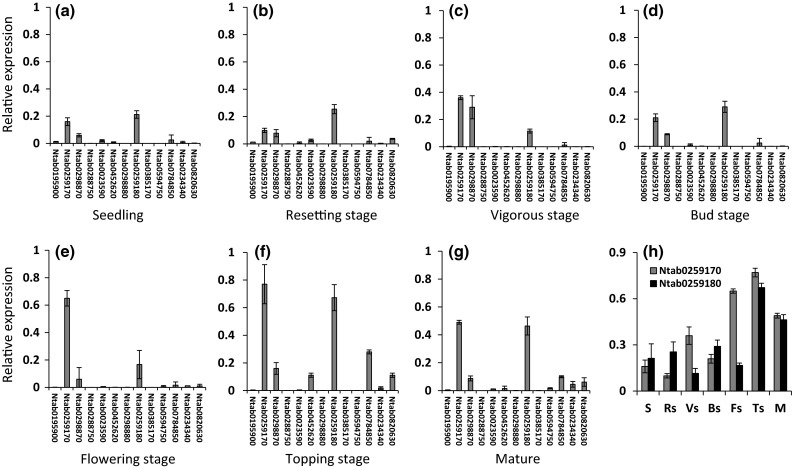
RT-qPCR analysis of *NtSus* genes at various developmental stages of tobacco leaves (**a**–**g**). (**h**) Relative expression levels of Ntab0259170 and Ntab0259180. *S* seedling, *Rs* resetting stage, *Vs* vigorous stage, *Bs* bud stage, *Fs* flower stage, *Ts* topping stage, *M* mature. The expression levels of the *NtSus* genes were related to the internal control *26S* gene. Values are mean ± SD of three independent biological replicates

Since sucrose affects cell division and vascular tissue differentiation in plant leaves, we further performed quantitative real-time RT-PCR to detect the relative expression levels of each *NtSus* gene at different developmental stages of tobacco leaves (Fig. 6). Transcripts of only three *NtSus* genes were detected in the leaves of seedlings and leaves of plants at the resetting stage, vigorous stage, bud stage, or flowering stage, whereas there were six genes expressed in leaves at the topping and mature stages (Fig. 6g). In detail, Ntab0259170, Ntab0298870, and Ntab0259180 were ubiquitously expressed at all leaf growth stages tested. Transcription levels of Ntab0259170 and Ntab0259180 slightly increased during the course of leaf development, and reached maximal expression levels at the topping stage (Fig. 6h), indicating that these two genes encode isozymes that catalyze key aspects of sucrose metabolism in leaves in late development stages.

**Fig. 7:**
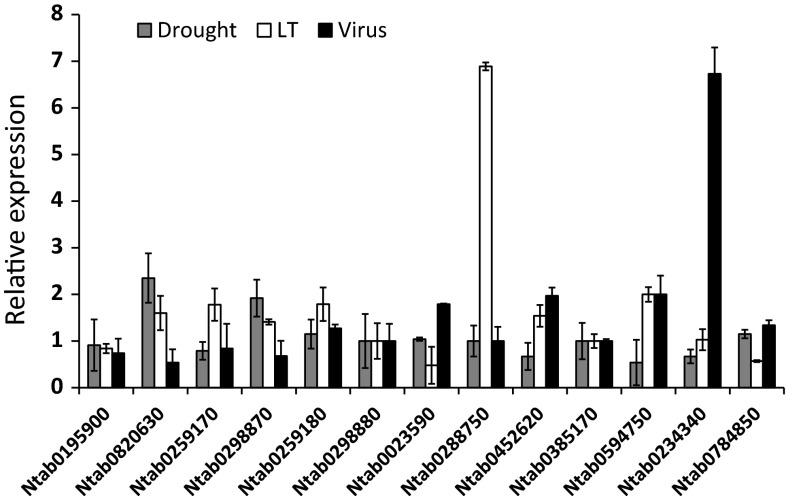
Expression profiles of the *NtSus* genes in response to drought, low temperature (LT), and virus treatments. Values are mean ± SD of three independent biological replicates

To determine whether the *NtSus* genes were involved in stress resistance, we measured their transcript levels under drought, low temperature, and virus treatments. As is shown in Fig. 7, when suffering drought stress, the expression levels of all of the *NtSus* genes were similar to those of the control, except for Ntab0820630 and Ntab0298870, for which the transcript levels were up-regulated approximately twofold. Moreover, the expression level of Ntab0288750 transcripts was sixfold higher under low-temperature treatment, while the expression levels of transcripts of the other *NtSus* genes were merely slightly increased (less than twofold) under this treatment. Similarly, when inoculated with a virus, the transcript level of Ntab0234340 increased much more dramatically than did the levels of the other *NtSus* genes. Taken together, our results show that different environmental stresses could induce the transcription of different *NtSus* genes, indicating diverse functions of the *NtSus* genes in plant responses to stress.

## Discussion

Comparative genome approaches have been used to analyze many *Sus* gene families in various plant species, including Arabidopsis, rice, maize, popular, and cotton. In the present study, benefiting from sequencing efforts of the whole tobacco genome conducted by the China Tobacco Gene Research Center (manuscript under review), we identified 14, 6, and 7 *Sus* genes from *N.tabacum*, *N.sylvestris,* and *N.tomentosiformis*, respectively. These tobacco Sus family members shared high levels of similarity in both their nucleotide and amino acid sequences; we named these as Sus1 to Sus7, according to distinct molecular signatures. We further analyzed their molecular structures, evolutionary relationships, and expression patterns in various tobacco plant materials. Therefore, this study provides a foundation for understanding the putative functions of these genes in various growth and developmental processes in tobacco.

### Evolutionary conservation and divergence among tobacco *Sus* genes

The Sus isozymes in plants examined to date are encoded by a small, multi-gene family. Comprehensive analysis of this multi-gene family, including consideration of its exon/intron gene structures, phylogeny, and conserved motifs, allows researchers to generalize and predict the possible genetic and evolutionary relationships among uncharacterized members of this gene family, as well as to predict their possible functions. Previous studies of the molecular structures and phylogenetic relationships of plant Sus sequences divided them into three major groups, namely Sus1, SusA, and New Group (Horst et al. 2007; Hirose et al. 2008). This classification was corroborated in subsequent studies, and these three groups were renamed as the Sus I, Sus II, and Sus III groups, respectively (Zhang et al. 2011; Chen et al. 2012; Zou et al. 2013). Later, the Sus I group was divided into a monocot subgroup and a eudicot subgroup, as these two subgroups were obviously separate from each other in phylogenetic trees. The Sus II and Sus III groups were then considered as mix group 1 (monocot and eudicot group 1) and mix group 2, according to the categories of group members (An et al. 2014). In our study, we performed phylogenetic analysis of the Sus homologs from 3 tobacco species and those of eight other plant species, and found that the tobacco Sus family had at least one gene in three separate groups. The four tobacco *Sus* genes in the eudicot group were divided into two subgroups, among which, tobacco *Sus1* and *Sus2* clustered together, while *Sus3* and *Sus4* genes were apparently apart from *Sus1*, *Sus2*, and *Sus* homologs of Arabidopsis and cotton. This result suggests that a gene duplication event that generated tobacco *Sus3* and *Sus4* occurred after the separation of monocots and eudicots species, but before the divergence of Solanaceae/Arabidopsis/Gossypium. Moreover, the generation of the tobacco *Sus1* and *Sus2* genes probably took place after the separation of Solanaceae/Arabidopsis/Gossypium, but before the divergence of Solanaceae. In the Sus II and Sus III groups, the tobacco *Sus5*, *Sus6,* and *Sus7* genes all closely clustered together with Solanaceae *Sus* genes (Fig. 4), indicating the generation of these three genes were also before the divergence of Solanaceae. Thus, tobacco *Sus3* and *Sus4* were older than the other tobacco *Sus* genes. Further, we analyzed an additional phylogenetic tree of the Solanaceae *Sus* genes (Suppl. Fig. S6), in which the *Sus* genes were apparently divided into five groups. *NtSus3* and *NtSus4* belonged to group II, which also contained genes from pepper (*CaSus4*) and tomato (*SolySus4*). The phylogenetic tree also showed that the formation of *NtSus4* occurred prior to that of *NtSus3*, *CaSus4*, and *SolySus4*. So, it can be concluded that the oldest gene in the tobacco *Sus* gene family is *NtSus4*, and this was lost from *N. sylvestris* during evolution.

Since gene exon structures are typically highly conserved among homologous genes in duplicated gene families (Frugoli et al. 1998), analysis of exon/intron structures can provide clues to reveal the evolutionary history of gene families (Lecharny et al. 2003). Previous studies have shown that there are 14 conserved introns in most of the *Sus* genes in the major three Sus groups (Sus I, Sus II and Sus III), leading to the speculation that the divergence of the three progenitors predated the segregation of monocot and eudicot species (Tang et al. 2008). Moreover, the genes of the Sus I monocot group contain more introns than the genes of the Sus I eudicot group; this led to the supposition that intron loss events took place at least twice in the evolution of the eudicot *Sus* genes under selection pressure (Chen et al. 2012). The exon/intron structures of the *Sus* genes in the Sus II group are similar in monocots, eudicots, and the putative ancestral *Sus* genes, indicating a relatively slower evolutionary rate for genes in this group. The *Sus* genes in the Sus III group share a remarkable feature: they have either an additional exon or a longer exon in their 3′ regions (Chen et al. 2012). In the present study, we evaluated the exon/intron structures of the tobacco *Sus* genes, and identified 14 conserved introns (Figs. 2, 3). Tobacco *Sus* genes in the Sus I monocot group and the Sus I eudicot group contained fewer conserved introns than genes in the other two groups. This was particularly obvious for the *Sus4* gene, for which there are 10 introns in *N.tabacum*, and 9 in *N.tomentosiformis*, suggesting this gene might be under high selection pressure. In contrast, the *NtSus5* genes of the Sus II group likely had slower evolutionary rates, as they contained the 14 conserved introns, consistent with former inference (Chen et al. 2012). Moreover, we observed the previously noted feature in the 3′ regions of tobacco *Sus6* and *Sus7* genes, which further supports the idea that there might have been ectopic recombination between the progenitor of the Sus II group and a sequence with at least two introns prior to the divergence of monocots and eudicots.

We propose an evolutionary history of the tobacco *Sus* genes based on our phylogenetic analysis and our analysis of exon/intron structures. Before the split of monocots and eudicots, duplication of the ancestral gene gave rise to the three progenitors of the three Sus groups with 14 conserved introns. Two of the three precursors underwent independent evolution and finally retained one single gene in tobacco (*Sus5*) in the Sus II group and two genes (*Sus6* and *Sus7*) in the Sus III group. After the divergence of monocots and eudicots, duplication of the *Sus* precursor generated *Sus3* and *Sus4*, whereas *Sus1* and *Sus2* were produced after differentiation within the Solanaceae (Fig. 4). Although different in evolutionary time and trajectory, tobacco *Sus2* and *Sus3* are closely linked to each other in the genomes of both the diploid and tetraploid tobacco species. Intriguingly, the expression pattern of *NtSus2* (Ntab0259170) was similar to that of *NtSus3* (Ntab0259180) in various developmental stages of tobacco leaves (Fig. 6). These findings may prove useful in tobacco breeding efforts to improve leaf development. Moreover, each *Sus* gene in *N.tabacum* (tetraploid tobacco) contained two paralogs, of which one shared high similarity with orthologs in *N.sylvestris* or *N.tomentosiformis*, further corroborating the idea that tetraploid tobacco was formed by a cross between these two diploid species, followed by chromosome reduplication.

### Divergence in *NtSus* gene expression patterns

To differentiate new organs or adapt to various environments, plants have to make evolutionary changes in protein property and/or in the expression patterns of particular genes (Gu et al. 2003; Flagel and Wendel 2009). Analysis of gene expression patterns can be used to some extent to predict the molecular functions of genes involved in different physiological processes. To date, although the expression patterns of *Sus* genes have been characterized in detail in several plant species, such as Arabidopsis, rice, cotton, poplar, and rubber tree (Baud et al. 2004; Hirose et al. 2008; Chen et al. 2012; Zou et al. 2013; An et al. 2014; Xiao et al. 2014), there have been no detailed analyses of the expression patterns of *Sus* genes in tobacco.

Sus isozymes have been shown to participate in the regulation of sink strength in plants (Fu and Park 1995; Zrenner et al. 1995; Chourey et al. 1998; Tang and Sturm 1999; Barratt et al. 2001). In the present study, we first measured the transcription levels of *NtSus* genes in different tissues, and found that no two *NtSus* genes shared identical expression patterns (Fig. 5). No *NtSus* gene was expressed exclusively in a single tissue, and none of the tissues had expression of only a single *NtSus* gene. These results suggest that the functions of *NtSus* genes are diversified and yet partially overlap. Our results further demonstrated that the expression levels of most of the *NtSus* genes was higher in sink tissues, such as roots, buds, flowers (including sepals, stamens, and pistils), and seeds than in source tissues (Fig. 5). Most *NtSus* genes were not expressed, or were expressed at low levels along the course of development of leaves, whereas transcription of Ntab0259170, Ntab0259180, and Ntab0298870 could always be detected (Fig. 6). Therefore, these three genes might be key regulators of sucrose metabolism in leaves, which are the most economically valuable parts of tobacco plants. We compared the transcription levels of Ntab0259170 and Ntab0259180 in leaves at different developmental stages (Fig. 6h). The low expression levels of the two *NtSus* genes (Ntab0259170 and Ntab0259180) in young leaves were consistent with the idea that *Sus* genes are mainly expressed in sink tissues, rather than in source tissues (Turner and Turner 1975). However, after topping (a typical agronomic practice in tobacco production), the transcription levels of Ntab0259170 and Ntab0259180 were significantly up-regulated in leaves, as was the expression of Ntab0452620 and Ntab0820630, which were hardly detected in previous stages (Fig. 6f). The increased expression of these genes might be caused by ‘role conversion’ of leaves in tobacco plants before and after topping (i.e., from source tissue to sink tissue). In mature leaves, it is known that photosynthetic capacity and metabolic activity decreases (Gan and Amasino 1997), possibly resulting in relatively lower expression levels of Ntab0259170 and Ntab0259180 genes.

Sucrose synthases have been supposed to participate in plant resistance to various environmental stresses. For instance, transcription of the *AtSus1* gene can be induced by cold or mannitol treatment in Arabidopsis. *AtSus3* is used as a molecular genetic marker of dehydration (Baud et al. 2004). Similarly, both low temperature and drought stress can conspicuously induce the expression of two barley *Sus* genes (*HvSs1* and 3) and one rubber tree *Sus* gene (*HbSus5*) (Barrero et al. 2011; Xiao et al. 2014). The higher expression levels of *Sus* genes may relate to meeting the increased glycolytic demand that occurs under abiotic stresses (Kleines et al. 1999). The expression patterns of the *NtSus* genes differed from each other in the three experiments (Fig. 7). In detail, under the low temperature and virus treatments, the transcription levels of Ntab0288750 and Ntab0234340 were significantly up-regulated (over sixfold), respectively, suggesting that these genes might encode key glycolysis enzymes under cold and virus infection conditions. There were two genes (Ntab0820630 and Ntab0298870) that showed increased transcription levels (around twofold) under drought treatment, while those of the other *NtSus* genes were similar to the control. This result remains to be confirmed as biologically relevant, as treatment with PEG2000 does not fully simulate drought conditions (Verslues et al. 2006).

The sequencing of the whole tobacco genome conducted by the China Tobacco Gene Research Center provides the plant biology community with a wealth of new information for functional genomics. Benefitting from this sequencing database (manuscript under review), we identified and characterized the tobacco *Sus* gene family, considering the physical structures, evolutionary histories, and expression patterns of these gens in different tobacco plant materials. Our findings will be helpful for efforts to further understand the functions of these important enzymes in various growth and developmental processes in tobacco.

#### *Author contribution*

ZW and JY conceived and designed the experiments. ZW conducted experiments and prepared initial draft manuscript. PW, MW, FL, ZL, JZ and XX performed RT-PCR and RT-qPCR. JY, AC, YX and PC performed partial bioinformatics analysis. FL and JY provided suggestions for the article writing and modified the manuscript. All authors in this study read and approved the manuscript.

## Electronic supplementary material

Supplementary material 1 Predicted amino acid sequences of the 14 *NtSus* genes. Identical amino acids are shaded; gaps are indicated by dots. The boxes indicate putative conserved serine residues predicted to be recognized by Ser/Thr protein kinase for phosphorylation. The dashed line shows the characteristic sucrose synthase domain, and the solid line indicates the glycosyl transferase domain (PPTX 240 kb)

Supplementary material 2 Location of *Sus* gene family members on the *Nicotiana tomentosiformis* chromosomes. Scale represents a 20.0-Mb chromosomal distance. Chromosome sizes (Mb) and numbers are indicated at the bottom end of each scaffold (PPTX 53 kb)

Supplementary material 3 Location of *Sus* gene family members on the *Nicotiana sylvestris* chromosomes. Scale represents a 20.0-Mb chromosomal distance. Chromosome sizes (Mb) and numbers are indicated at the bottom end of each scaffold (PPTX 53 kb)

Supplementary material 4 Exon/Intron structural organization of the *NsylSus* and the *NtomSus* genes. Black boxes denote exons within coding regions, and the lines connecting them represent introns. Numbers in boxes or above lines represent the sizes (bp) of corresponding exons or introns, respectively. The 5′ and 3′ untranslated regions (UTRs) are represented by blank boxes (PPTX 121 kb)

Supplementary material 5 Distribution of conserved motifs in amino acid sequences of tobacco Sus proteins (PPTX 119 kb)

Supplementary material 6 Phylogenetic analysis of Solanaceae Sus isoforms (PPTX 90 kb)

Supplementary material 7 (PPTX 88 kb)

Supplementary material 8 (PPTX 98 kb)

## Abbreviations

Sus: Sucrose synthase
Inv: Invertase
UDP: Uridine diphosphate
PEG: Polyethylene glycol
gDNA: Genomic DNA
CDS: Coding sequence
Mw: Molecular weight
pI: Isoelectric point
