# Supplementary figures and images for: Analysis of the sucrose synthase gene family in tobacco: structure, phylogeny, and expression patterns
Source: Planta. 2015 Apr 19;242(1):153–66. doi: 10.1007/s00425-015-2297-1 (PMC4471321; doi:10.1007/s00425-015-2297-1)

## Slide 1
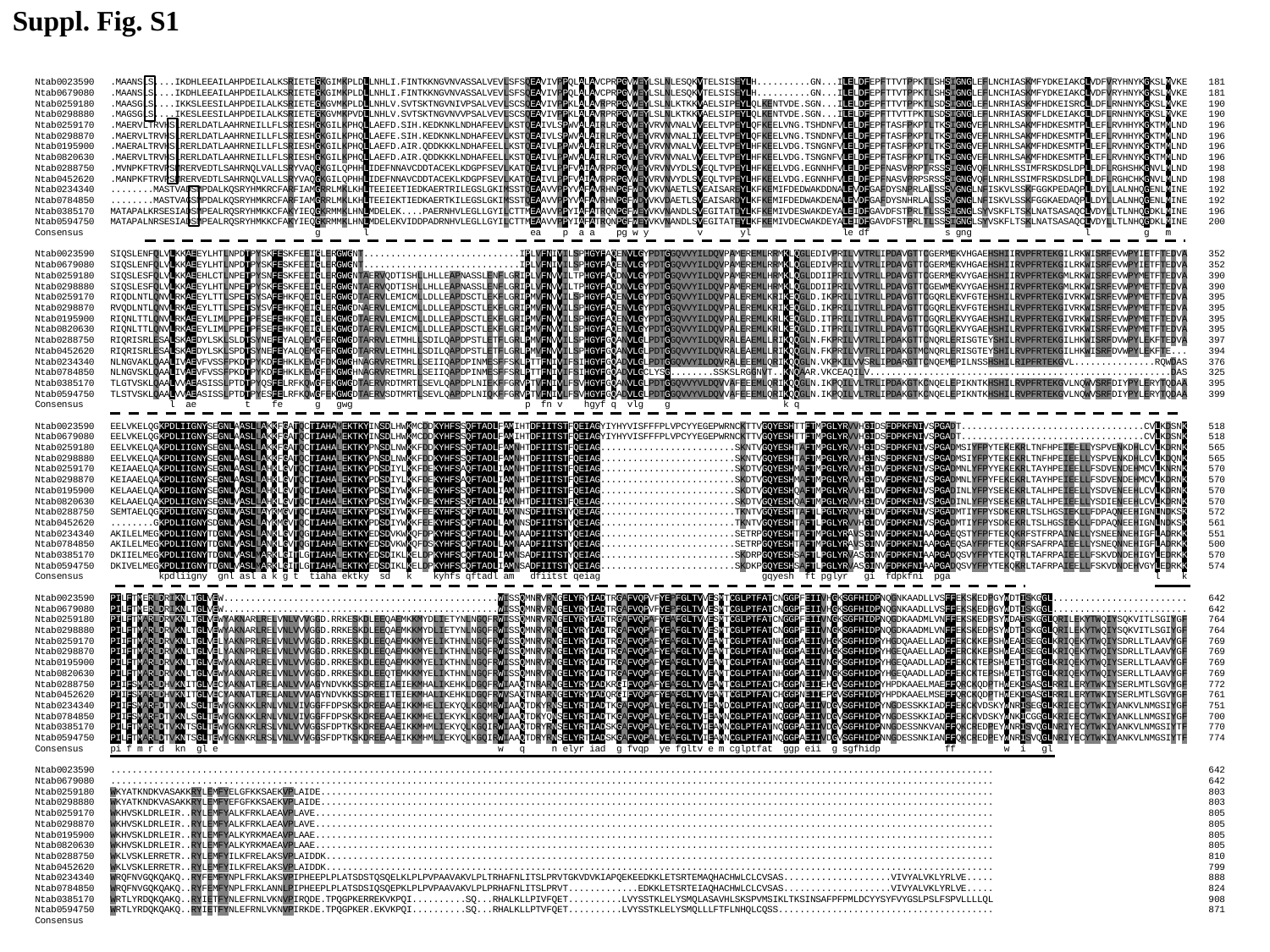

Suppl. Fig. S1

Supplement: Supplementary file 1 — Supplementary material 1 Predicted amino acid sequences of the 14 NtSus genes. Identical amino acids are shaded; gaps are indicated by dots. The boxes indicate putative conserved serine residues predicted to be recognized by Ser/Thr protein kinase for phosphorylation. The dashed line shows the characteristic sucrose synthase domain, and the solid line indicates the glycosyl transferase domain (PPTX 240 kb) [file 425_2015_2297_MOESM1_ESM.pptx]

## Slide 1
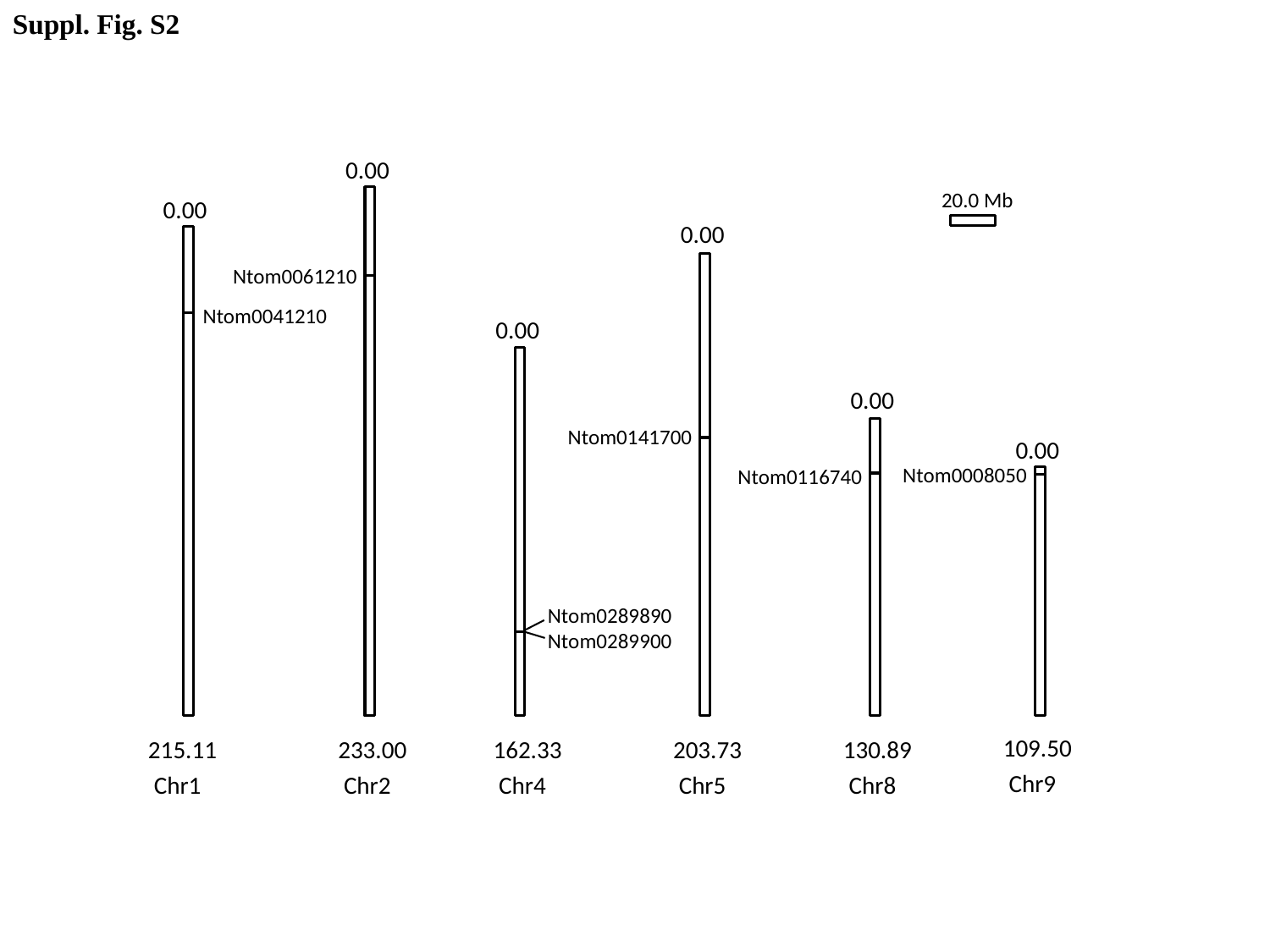

Suppl. Fig. S2
0.00
Ntom0061210
233.00
Chr2
20.0 Mb
0.00
Ntom0041210
215.11
Chr1
0.00
Ntom0141700
203.73
Chr5
0.00
Ntom0289890
Ntom0289900
162.33
Chr4
0.00
Ntom0116740
130.89
Chr8
0.00
Ntom0008050
109.50
Chr9

Supplement: Supplementary file 2 — Supplementary material 2 Location of Sus gene family members on the Nicotiana tomentosiformis chromosomes. Scale represents a 20.0-Mb chromosomal distance. Chromosome sizes (Mb) and numbers are indicated at the bottom end of each scaffold (PPTX 53 kb) [file 425_2015_2297_MOESM2_ESM.pptx]

## Slide 1
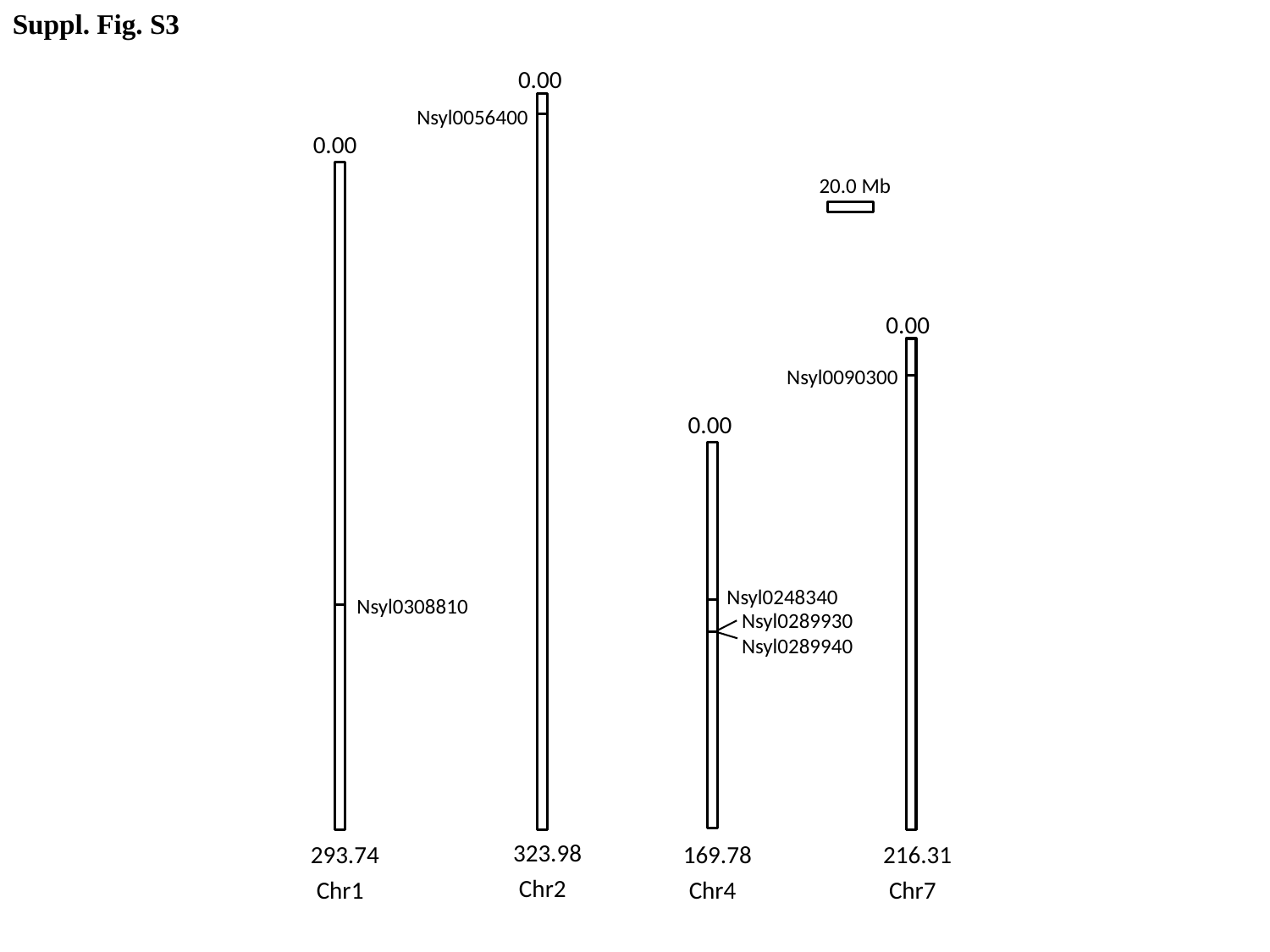

Suppl. Fig. S3
0.00
Nsyl0056400
323.98
Chr2
0.00
Nsyl0308810
293.74
Chr1
20.0 Mb
0.00
Nsyl0090300
216.31
Chr7
0.00
Nsyl0248340
Nsyl0289930
Nsyl0289940
169.78
Chr4

Supplement: Supplementary file 3 — Supplementary material 3 Location of Sus gene family members on the Nicotiana sylvestris chromosomes. Scale represents a 20.0-Mb chromosomal distance. Chromosome sizes (Mb) and numbers are indicated at the bottom end of each scaffold (PPTX 53 kb) [file 425_2015_2297_MOESM3_ESM.pptx]

## Slide 1
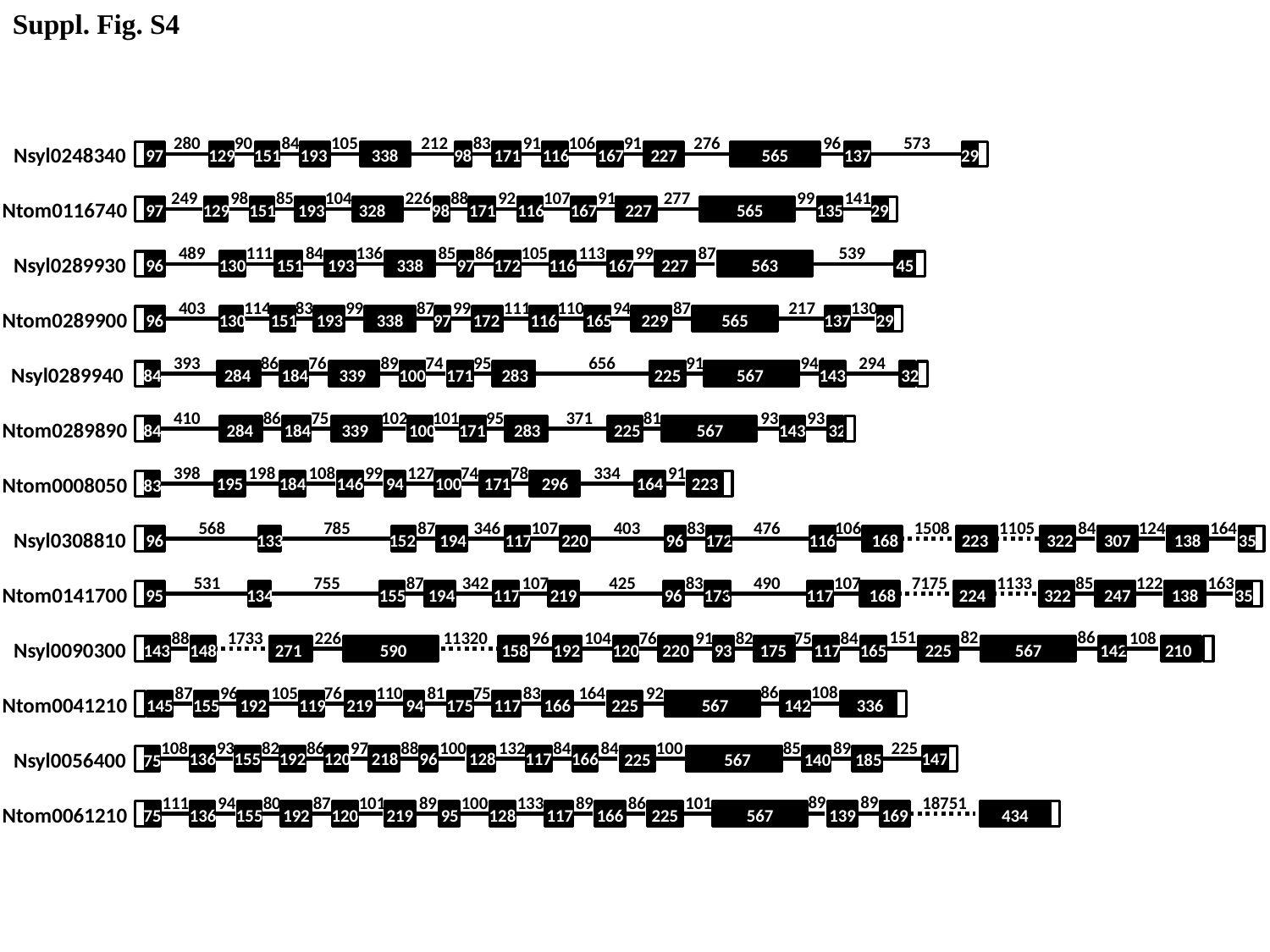

Suppl. Fig. S4
Nsyl0248340
573
106
280
90
84
105
212
83
91
91
276
96
167
29
97
129
151
193
338
98
171
116
227
565
137
Ntom0116740
141
107
249
98
85
104
226
88
92
91
277
99
167
29
97
129
151
193
328
98
171
116
227
565
135
Nsyl0289930
113
489
111
84
136
85
86
105
99
87
539
167
96
130
151
193
338
97
172
116
227
563
45
Ntom0289900
130
110
403
114
83
99
87
99
111
94
87
217
165
29
96
130
151
193
338
97
172
116
229
565
137
Nsyl0289940
393
86
76
89
74
95
656
91
94
294
84
284
184
339
100
171
283
225
567
143
32
Ntom0289890
410
86
75
102
101
95
371
81
93
93
84
284
184
339
100
171
283
225
567
143
32
Ntom0008050
398
198
108
99
127
74
78
334
91
195
184
146
94
100
171
296
164
166
223
83
Nsyl0308810
84
124
164
476
568
785
87
346
107
403
83
106
1508
1105
307
138
116
35
96
133
152
194
117
220
96
172
168
223
322
Ntom0141700
85
122
163
490
531
755
87
342
107
425
83
107
7175
1133
247
138
117
35
95
134
155
194
117
219
96
173
168
224
322
Nsyl0090300
82
151
86
108
91
88
1733
226
11320
96
104
76
82
75
84
225
142
93
210
143
148
271
590
158
192
120
220
175
117
165
567
Ntom0041210
86
108
75
87
96
105
76
110
81
83
164
92
142
117
336
145
155
192
119
219
94
175
166
225
567
Nsyl0056400
108
93
82
86
97
88
100
132
84
225
84
100
85
89
136
155
192
120
218
96
128
117
166
147
225
567
140
185
75
Ntom0061210
89
89
18751
133
111
94
80
87
101
89
100
89
86
101
139
169
117
434
75
136
155
192
120
219
95
128
166
225
567

Supplement: Supplementary file 4 — Supplementary material 4 Exon/Intron structural organization of the NsylSus and the NtomSus genes. Black boxes denote exons within coding regions, and the lines connecting them represent introns. Numbers in boxes or above lines represent the sizes (bp) of corresponding exons or introns, respectively. The 5′ and 3′ untranslated regions (UTRs) are represented by blank boxes (PPTX 121 kb) [file 425_2015_2297_MOESM4_ESM.pptx]

## Slide 1
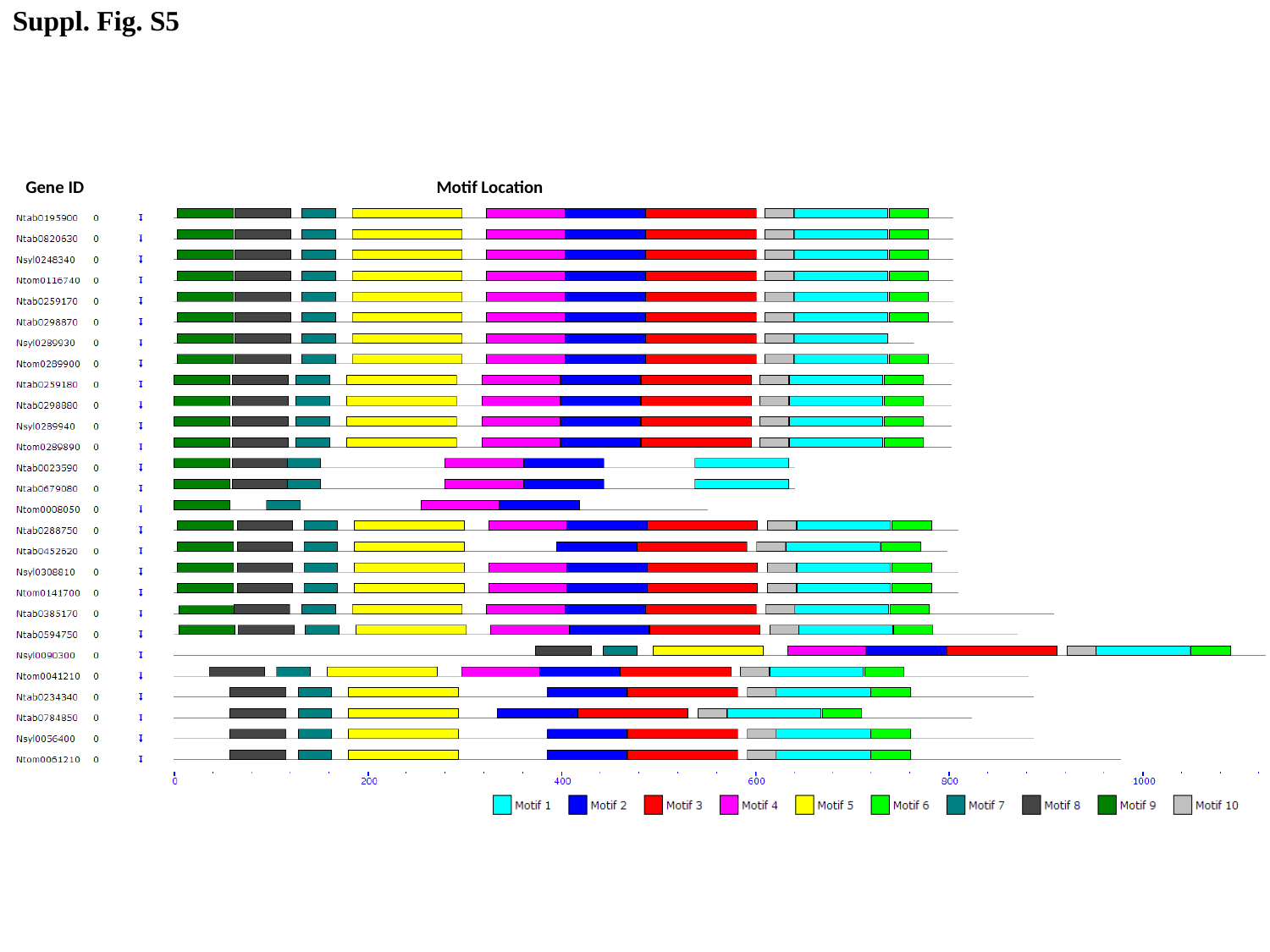

Suppl. Fig. S5
Gene ID
Motif Location

Supplement: Supplementary file 5 — Supplementary material 5 Distribution of conserved motifs in amino acid sequences of tobacco Sus proteins (PPTX 119 kb) [file 425_2015_2297_MOESM5_ESM.pptx]
