# Supplementary material for: Analysis of the sucrose synthase gene family in tobacco: structure, phylogeny, and expression patterns
Source: Planta. 2015 Apr 19;242(1):153–66. doi: 10.1007/s00425-015-2297-1 (PMC4471321; doi:10.1007/s00425-015-2297-1)
Supplement: Supplementary file 6 — Supplementary material 6 Phylogenetic analysis of Solanaceae Sus isoforms (PPTX 90 kb) [file 425_2015_2297_MOESM6_ESM.pptx]

## Slide 1
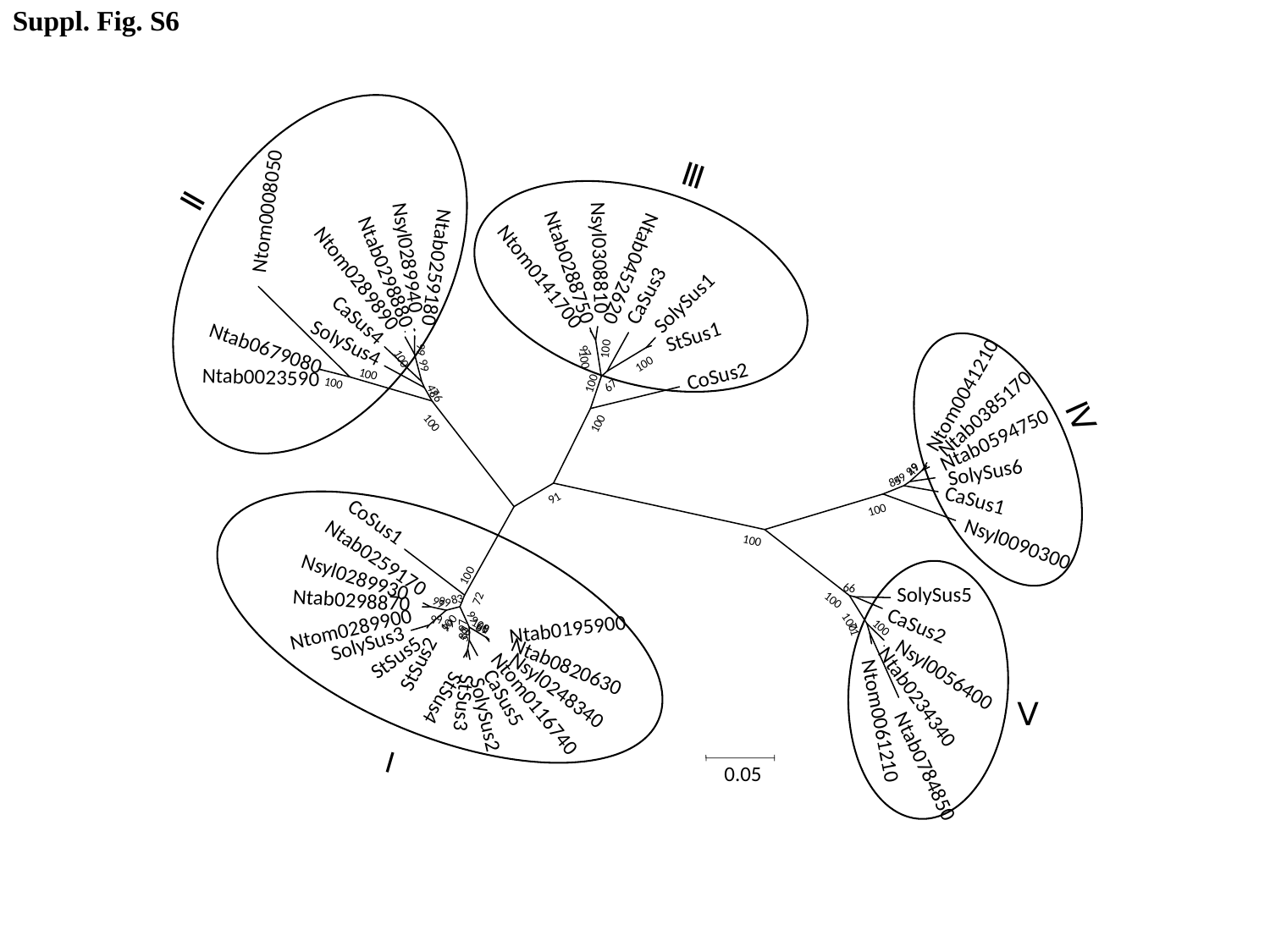

Suppl. Fig. S6
Ntom0008050
Nsyl0289940
Nsyl0308810
Ntab0288750
Ntab0259180
Ntab0452620
Ntab0298880
Ntom0141700
Ntom0289890
CaSus3
SolySus1
CaSus4
StSus1
SolySus4
Ntab0679080
100
99
97
100
100
100
99
CoSus2
Ntab0023590
100
100
100
67
Ntom0041210
48
76
Ntab0385170
100
100
Ntab0594750
SolySus6
99
49
59
84
CaSus1
91
100
CoSus1
Nsyl0090300
100
Ntab0259170
Nsyl0289930
100
66
SolySus5
Ntab0298870
72
83
100
99
99
99
99
CaSus2
100
100
Ntom0289900
Ntab0195900
97
100
99
100
68
61
71
83
58
SolySus3
StSus5
StSus2
Ntab0820630
Nsyl0056400
Nsyl0248340
Ntab0234340
CaSus5
StSus4
StSus3
Ntom0116740
SolySus2
Ntom0061210
Ntab0784850
0.05
Ⅲ
Ⅱ
Ⅳ
Ⅴ
Ⅰ
