# Supplementary material for: Analysis of the sucrose synthase gene family in tobacco: structure, phylogeny, and expression patterns
Source: Planta. 2015 Apr 19;242(1):153–66. doi: 10.1007/s00425-015-2297-1 (PMC4471321; doi:10.1007/s00425-015-2297-1)
Supplement: Supplementary file 7 — Supplementary material 7 (PPTX 88 kb) [file 425_2015_2297_MOESM7_ESM.pptx]

## Slide 1
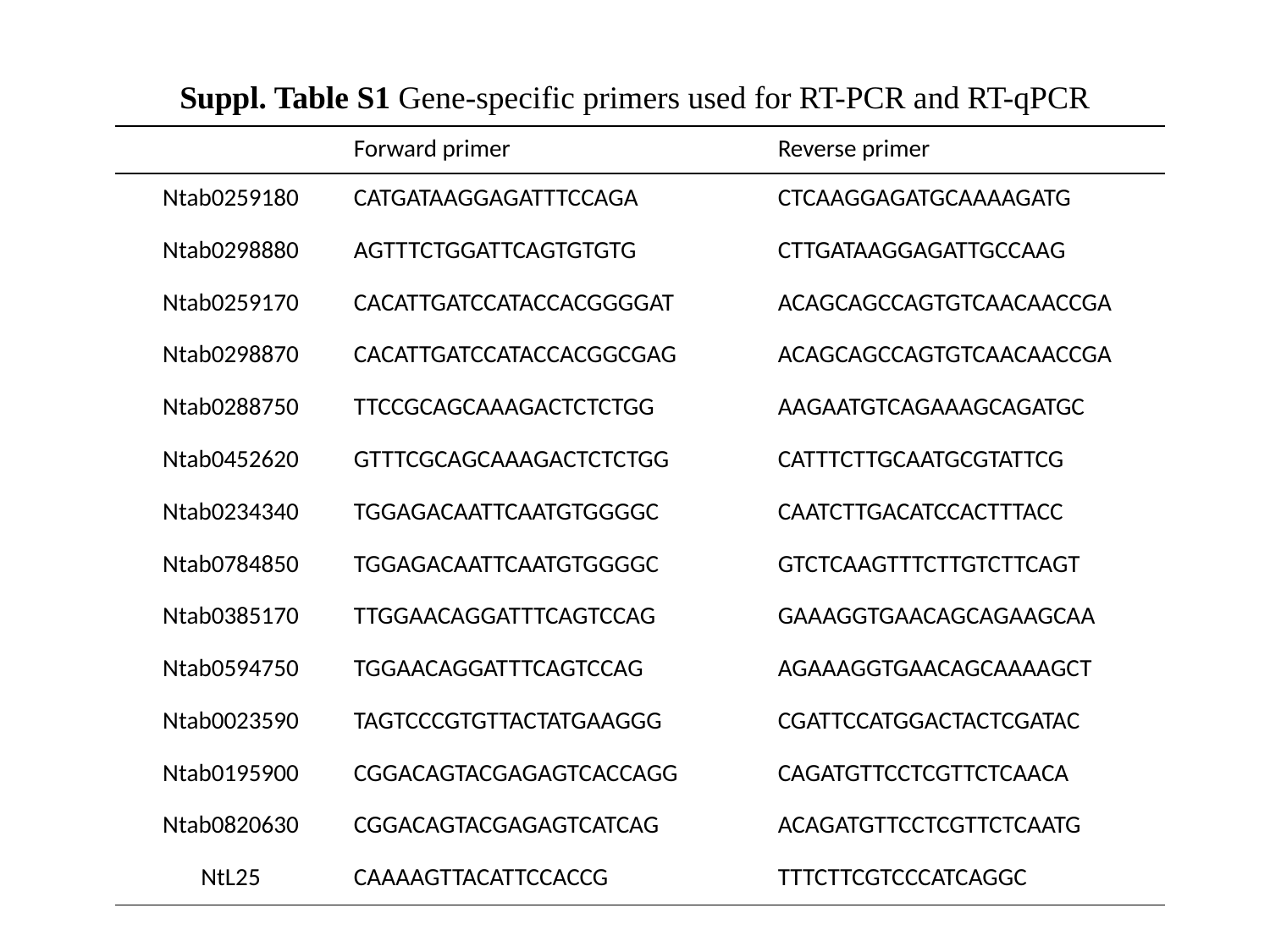

Suppl. Table S1 Gene-specific primers used for RT-PCR and RT-qPCR
| | Forward primer | Reverse primer |
| --- | --- | --- |
| Ntab0259180 | CATGATAAGGAGATTTCCAGA | CTCAAGGAGATGCAAAAGATG |
| Ntab0298880 | AGTTTCTGGATTCAGTGTGTG | CTTGATAAGGAGATTGCCAAG |
| Ntab0259170 | CACATTGATCCATACCACGGGGAT | ACAGCAGCCAGTGTCAACAACCGA |
| Ntab0298870 | CACATTGATCCATACCACGGCGAG | ACAGCAGCCAGTGTCAACAACCGA |
| Ntab0288750 | TTCCGCAGCAAAGACTCTCTGG | AAGAATGTCAGAAAGCAGATGC |
| Ntab0452620 | GTTTCGCAGCAAAGACTCTCTGG | CATTTCTTGCAATGCGTATTCG |
| Ntab0234340 | TGGAGACAATTCAATGTGGGGC | CAATCTTGACATCCACTTTACC |
| Ntab0784850 | TGGAGACAATTCAATGTGGGGC | GTCTCAAGTTTCTTGTCTTCAGT |
| Ntab0385170 | TTGGAACAGGATTTCAGTCCAG | GAAAGGTGAACAGCAGAAGCAA |
| Ntab0594750 | TGGAACAGGATTTCAGTCCAG | AGAAAGGTGAACAGCAAAAGCT |
| Ntab0023590 | TAGTCCCGTGTTACTATGAAGGG | CGATTCCATGGACTACTCGATAC |
| Ntab0195900 | CGGACAGTACGAGAGTCACCAGG | CAGATGTTCCTCGTTCTCAACA |
| Ntab0820630 | CGGACAGTACGAGAGTCATCAG | ACAGATGTTCCTCGTTCTCAATG |
| NtL25 | CAAAAGTTACATTCCACCG | TTTCTTCGTCCCATCAGGC |
