# Supplementary material for: Analysis of the sucrose synthase gene family in tobacco: structure, phylogeny, and expression patterns
Source: Planta. 2015 Apr 19;242(1):153–66. doi: 10.1007/s00425-015-2297-1 (PMC4471321; doi:10.1007/s00425-015-2297-1)
Supplement: Supplementary file 8 — Supplementary material 8 (PPTX 98 kb) [file 425_2015_2297_MOESM8_ESM.pptx]

## Slide 1
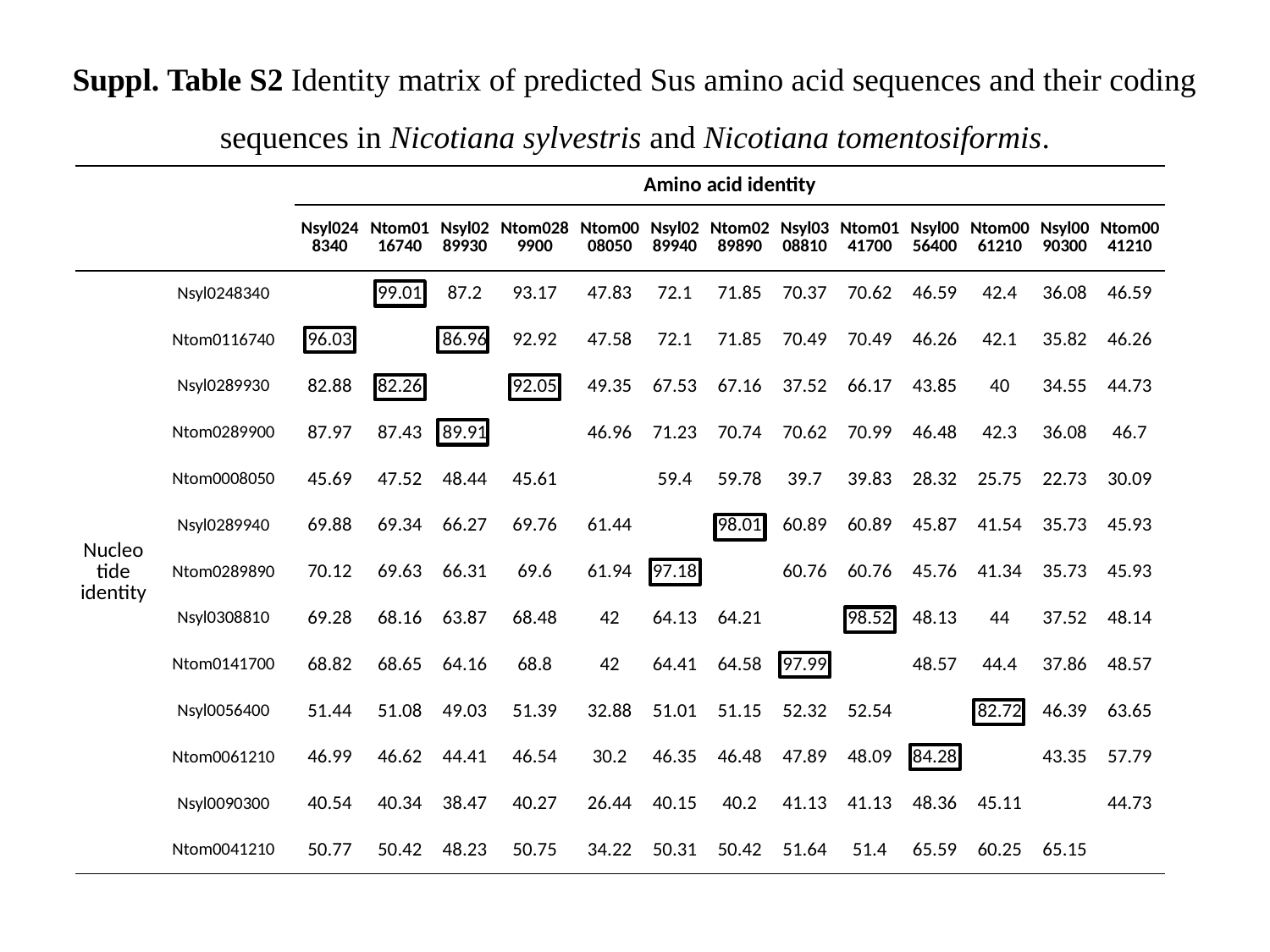

Suppl. Table S2 Identity matrix of predicted Sus amino acid sequences and their coding sequences in Nicotiana sylvestris and Nicotiana tomentosiformis.
| | | Amino acid identity | | | | | | | | | | | | |
| --- | --- | --- | --- | --- | --- | --- | --- | --- | --- | --- | --- | --- | --- | --- |
| | | Nsyl0248340 | Ntom0116740 | Nsyl0289930 | Ntom0289900 | Ntom0008050 | Nsyl0289940 | Ntom0289890 | Nsyl0308810 | Ntom0141700 | Nsyl0056400 | Ntom0061210 | Nsyl0090300 | Ntom0041210 |
| Nucleotide identity | Nsyl0248340 | | 99.01 | 87.2 | 93.17 | 47.83 | 72.1 | 71.85 | 70.37 | 70.62 | 46.59 | 42.4 | 36.08 | 46.59 |
| | Ntom0116740 | 96.03 | | 86.96 | 92.92 | 47.58 | 72.1 | 71.85 | 70.49 | 70.49 | 46.26 | 42.1 | 35.82 | 46.26 |
| | Nsyl0289930 | 82.88 | 82.26 | | 92.05 | 49.35 | 67.53 | 67.16 | 37.52 | 66.17 | 43.85 | 40 | 34.55 | 44.73 |
| | Ntom0289900 | 87.97 | 87.43 | 89.91 | | 46.96 | 71.23 | 70.74 | 70.62 | 70.99 | 46.48 | 42.3 | 36.08 | 46.7 |
| | Ntom0008050 | 45.69 | 47.52 | 48.44 | 45.61 | | 59.4 | 59.78 | 39.7 | 39.83 | 28.32 | 25.75 | 22.73 | 30.09 |
| | Nsyl0289940 | 69.88 | 69.34 | 66.27 | 69.76 | 61.44 | | 98.01 | 60.89 | 60.89 | 45.87 | 41.54 | 35.73 | 45.93 |
| | Ntom0289890 | 70.12 | 69.63 | 66.31 | 69.6 | 61.94 | 97.18 | | 60.76 | 60.76 | 45.76 | 41.34 | 35.73 | 45.93 |
| | Nsyl0308810 | 69.28 | 68.16 | 63.87 | 68.48 | 42 | 64.13 | 64.21 | | 98.52 | 48.13 | 44 | 37.52 | 48.14 |
| | Ntom0141700 | 68.82 | 68.65 | 64.16 | 68.8 | 42 | 64.41 | 64.58 | 97.99 | | 48.57 | 44.4 | 37.86 | 48.57 |
| | Nsyl0056400 | 51.44 | 51.08 | 49.03 | 51.39 | 32.88 | 51.01 | 51.15 | 52.32 | 52.54 | | 82.72 | 46.39 | 63.65 |
| | Ntom0061210 | 46.99 | 46.62 | 44.41 | 46.54 | 30.2 | 46.35 | 46.48 | 47.89 | 48.09 | 84.28 | | 43.35 | 57.79 |
| | Nsyl0090300 | 40.54 | 40.34 | 38.47 | 40.27 | 26.44 | 40.15 | 40.2 | 41.13 | 41.13 | 48.36 | 45.11 | | 44.73 |
| | Ntom0041210 | 50.77 | 50.42 | 48.23 | 50.75 | 34.22 | 50.31 | 50.42 | 51.64 | 51.4 | 65.59 | 60.25 | 65.15 | |
